# Development of an Alcohol Dehydrogenase Biosensor for Ethanol Determination with Toluidine Blue O Covalently Attached to a Cellulose Acetate Modified Electrode

**DOI:** 10.3390/s100100748

**Published:** 2010-01-21

**Authors:** Şenol Alpat, Azmi Telefoncu

**Affiliations:** 1 Department of Chemistry Education, Dokuz Eylul University, 35150 Buca-Izmir, Turkey;; 2 Department of Biochemistry, Ege University, 35100 Bornova-Izmir, Turkey; E-Mail: azmi.telefoncu@ege.edu.tr

**Keywords:** TBO, alcohol dehydrogenase, biosensor, NADH regeneration, voltammetry

## Abstract

In this work, a novel voltammetric ethanol biosensor was constructed using alcohol dehydrogenase (ADH). Firstly, alcohol dehydrogenase was immobilized on the surface of a glassy carbon electrode modified by cellulose acetate (CA) bonded to toluidine blue O (TBO). Secondly, the surface was covered by a glutaraldehyde/bovine serum albumin (BSA) cross-linking procedure to provide a new voltammetric sensor for the ethanol determination. In order to fabricate the biosensor, a new electrode matrix containing insoluble Toluidine Blue O (TBO) was obtained from the process, and enzyme/coenzyme was combined on the biosensor surface. The influence of various experimental conditions was examined for the characterization of the optimum analytical performance. The developed biosensor exhibited sensitive and selective determination of ethanol and showed a linear response between 1 × 10^−5^ M and 4 × 10^−4^ M ethanol. A detection limit calculated as three times the signal-to-noise ratio was 5.0 × 10^−6^ M. At the end of the 20^th^ day, the biosensor still retained 50% of its initial activity.

## 1. Introduction

Ethanol has been widely used in medicine, biotechnology and the food industry over the years [1]. When ethanol concentration reaches toxic levels in fermentation and distillation, it causes inflammation and conjunctiva of the nasal mucous membrane and irritation of the skin. In addition, alcohol poisoning occurs at higher ethanol concentration levels. Therefore, ethanol analysis is of great importance on account of its toxicological and psychological effects [2–5]. Several analytical methods have been developed so far for the determination of ethanol and other aliphatic alcohols. Commonly used methods are based on chromatographic techniques [2,6,7] such as gas chromatography [8], high performance liquid chromatography [9] and capillary electrophoresis [10], Raman spectrometry [11], colorimetric methods and other analogous methods [12]. The methods involved have some disadvantages such as requiring expensive instrumentation, long analysis time and their rather complex systems. An alternative way for the sensitive and rapid determination of ethanol is using biosensors based on selective ethanol-converting enzymes [2,7,13–17]. Alcohol oxidase [2,4,7,18–21], NAD^+^-dependent alcohol dehydrogenase [1,3,22–25], alcohol oxidase-peroxidase coupled system [20,26] and PQQ-dependent alcohol dehydrogenases [27,28] have been used many times as bioselective compounds in ethanol biosensors. NAD^+^-dependent alcohol dehydrogenase is selective for primary aliphatic and aromatic alcohols [20]. Nicotinamide adenine dinucleotide (NAD^+^) and its reduced form (NADH), a product of the reaction between NAD^+^ and primary alcohols, are the key central charge carriers in living cells. NAD^+^ is a very important cofactor since it participates in enzymatic catalysis of more than 300 dehydrogenase enzymes [29,30]. NAD(P)-dependent dehydrogenases are widely used in bioprocesses and analytical applications [3,31,32]. In the past decades, a considerable amount of analytical research has been related to the electrochemistry of the NAD^+^/NADH redox couple in various electrodes [33]. However, only a limited number of electrochemical sensors based on dehydrogenases were reported because of the need for cofactors for regeneration [22]. As the direct electro-oxidation of NADH on conventional electrode materials requires high overpotentials, many efforts have been devoted to develop new efficient electrode materials [7,22,29,32,34]. In order to decrease the high overpotentials and to minimize the side reactions, various mediators immobilized on the electrode surface have been widely used so far [17,22,35–37]. The immobilization of mediators such as catechols, quinones [38–41], ferrocene [42], phenoxazines [43–47], phenothiazines [47–49], phenylendiamines, some conducting polymers electrodeposited on electrode surface [50–53] and conducting charge-transfer complexes [54] has been investigated extensively. The use of such mediators immobilized on an electrode coupled with dehydrogenases and their applications to biosensor development are also described [3,7,55,56].

As a mediator, Toluidine Blue O (TBO), a phenothiazine derivative, is commonly used for the oxidation and determination of NADH [22,31–34,36,37]. However, it has some disadvantages due to its small molecular size and water solubility [3,33,43,57,58]. Therefore, some investigations have been carried out over the years to solve the problem of its immobilization [33,57,58].

There are several methods in the literature for immobilization of ADH on an electrode surface [3]. For this purpose, the surface of glassy carbon electrode or nickel electrode is modified with different reagents, some of which are poly(neutral red), Nafion membrane, poly(indole-5-carboxylic acid) and hexacyanoferrate [59–61]. ADH, NAD^+^ and meldola blue (MB) are coimmobilized in polypyrrole film. The Michaelis-Menten constant for ADH has even been determined [59–61].

In the present work, we have described the preparation of a new electrode matrix. We used it in order to obtain insoluble TBO on the electrode surface via covalent linkage between a cellulose acetate membrane and TBO molecules. After, the new electrode matrix was used for the development of a biosensor for sensitive and selective ethanol determination based on alcohol dehydrogenase.

## 2. Experimental

### 2.1. Chemicals and reagents

Toluidine Blue O (TBO, Aldrich), Nafion (Fluka), glutaraldehyde (Sigma), bovine serum albumin (Biological Industries), 1,1′-carbonyldiimidazole (CDI, Fluka) and cellulose acetate (Aldrich) were used for the modification of the glassy carbon electrode (GCE). Alcohol dehydrogenase (ADH) (E.C.1.1.1.1) and all chemicals used for preparation of buffer solutions and alcohols and were purchased from E. Merck (Darmstadt, Germany). NADH and NAD^+^ were obtained from Merck. The other chemicals were analytical grade. All solutions used in the experiments were prepared just before their use.

### 2.2. Apparatus

Electrochemical measurements were conducted with a Metrohm 746 Trace Analyser and 747 VA stand instrument. All experiments were carried out with a conventional three-electrode system: the modified glassy carbon electrode as the working electrode, a platinum wire as the auxiliary electrode, and Ag/AgCl (saturated KCl) electrode as the reference. Internal diameter of glassy carbon electrode was 3.3 mm and the obtained peak current values were given as a nA/cm^2^. Differential pulse voltammetry (DPV) measurements were operated with a scan rate of 15 mV/s. The pulse amplitude was 50 mV, pulse time was 40 ms and measuring time was 20 ms. Ultra pure deionized water (18 M Ωcm^−1^) was obtained from a USF ELGA UHQ water purification system. The solution temperature was controlled with a thermostat (PolyScience).

### 2.3. Synthesis of cellulose acetate with covalently attached TBO on the electrode surface

**Scheme 1.**
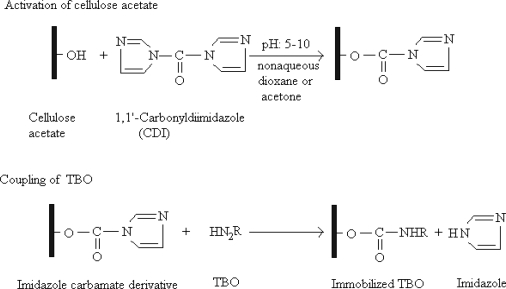
The procedure for immobilization of TBO on cellulose acetate.

Cellulose acetate (CA, 40.0 mg) was dissolved in dioxane (2.0 mL). Then, CDI (50.0 mg) was added to this cellulose acetate solution. This mixture was continuously stirred for 30 min at room temperature until the CDI was completely dissolved. In order to investigate the effect of CA membrane thickness on the electrode response, various ratios of cellulose acetate solutions were prepared (1.0%, 2.0%, 4.0%, 10.0% w/v). The reaction between cellulose acetate and CDI converted the hydroxyl groups on the cellulose acetate into imidazoylcarbamate derivatives [62,63]. The activated matrix is relatively stable to hydrolysis, but smoothly reacts with *N*-nucleophiles. Activated matrix (30.0 μL) was dropped onto a glassy carbon electrode and kept until the dioxane evaporated. Then, toluidine blue O solution (TBO, 100.0 μL) whose pH was adjusted with NaOH to 8.5, was dropped onto the electrode surface and kept overnight at 4 °C. Thus, a covalent ester bond was obtained by the linkage between TBO and cellulose acetate. The immobilization procedure is shown in Scheme 1. Following this period, the electrode was thoroughly rinsed with water at various times to remove non-bonded TBO from the electrode surface. The cleaning solutions were collected to determine the quantity of non-bounded TBO by UV-Vis spectrophotometry at 585 nm. According to the spectrophotometric data, bonded TBO was calculated. The modified electrode was rinsed carefully with 10^−5^ M glycine solution. In order to improve the permeability of the cellulose membranes, the TBO immobilized electrodes were then hydrolyzed in a 0.07 M KOH solution for 50 min.

### 2.4. Biosensor preparation

Ten mM NADH solution (50 μL) was dropped on the CA-TBO-modified glassy carbon electrode (CA-TBO/GC) surface. Alcohol dehydrogenase (200 U/mg, 1 mg) was dissolved in 50 mM phosphate buffer (PB, pH 7.0, 1 mL), then 50 μL was added on the electrode surface. 10.0% (w/v) of bovine serum albumin and 2.5% (w/v) of glutaraldehyde were prepared in 50 mM PB (pH 7.5) solution. Ten μL of bovine serum albumin and 10 μL of glutaraldehyde were dropped on the electrode surface, respectively. After that the excess of glutaraldehyde was rinsed off with water. In order to prevent the leakage of small molecules from the surface, the electrode surface was coated with 10 μL of 5.0% (w/v) Nafion solution.

### 2.5. Procedure

Voltammetric measurements were carried out in 10.0 mL of 50 mM PB (pH 7.0) prepared with ultra pure deionized water. The electrochemical cell containing the supporting electrolyte was purged by bubbling high-purity nitrogen for 300 s, before the measurements. The differential pulse voltammograms were respectively recorded anodic and cathodic directions between −400 mV–0 mV and 0 mV–(−400) mV, respectively.

### 2.6. Determination of ethanol in samples

Beer samples (25 mL) were diluted in a 250 mL flask with 50 mM PB phosphate buffer solution (pH 7.0). Voltammetric determination was carried out by applying the standard addition method. Diluted sample and standard ethanol solution (12.5 μL) were added to the voltammetric cell containing 10 mL of 50 mM PB phosphate buffer solution (pH 7.0).

## 3. Results and Discussion

### 3.1. Effect of cellulose acetate membrane thickness on the electrode response

Effect of the cellulose acetate membrane thickness on the electrode response was examined with various ratios of cellulose acetate to covalently attached TBO. Although the amount of bonded TBO in membranes containing 4.0% and 10.0% cellulose acetate was higher than in the others, the obtained peak currents were lower. On the other hand, the membranes contaning 1.0% and 2.0% cellulose acetate showed higher peak currents. In order to increase the permeability of the membranes containing 1.0% and 2.0% cellulose acetate, these membranes were hydrolyzed in 0.07 N KOH for 50 minutes. As a result, the diffusion barrier effect of membranes decreased and accordingly, NADH molecules could reach electrode surface easily due to the hydrolysis of acetyl groups in the cellulose acetate. After this hydrolysis procedure of the membranes, the peak currents increased dramatically. Especially, the peak current obtained from 2.0% w/v of cellulose acetate membrane showed a significant increase. Thus, the optimum cellulose acetate membrane ratio was chosen as 2.0% w/v (Table 1).

### 3.2. Electrochemical behaviour of CA-TBO/GC

There has always been an interest in electrochemical detection of NADH. The electrochemical oxidation of NADH to the corresponding oxidized form NAD^+^ at a bare electrode surface is highly irreversible and takes place at considerable overpotentials. One of the ways of achieving oxidation of NADH at lower potentials is to use a redox mediator, either present in solution or confined on the electrode surface. The electrocatalytic activity of the CA-TBO/GC electrode was investigated with cyclic and differential voltammetry. Upon addition of NADH into the solution, the voltammetric wave presents a clear enhancement of the anodic current. Therefore, it can be suggested that CA-TBO/GC acts efficiently as a catalyst for NADH oxidation.

**Figure 1.**
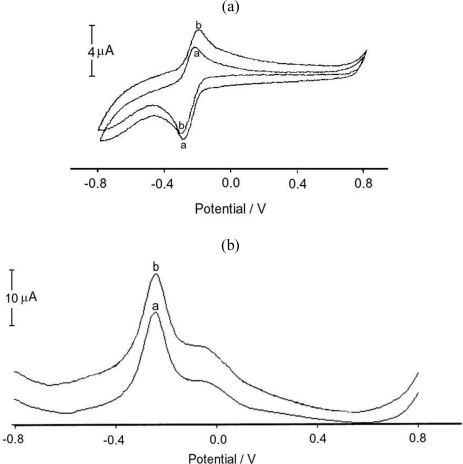
(a): Cyclic voltammograms obtained for a cellulose acetate modified with TBO glassy carbon electrode (a) in the absence of NADH and (b) in the presence of 1 mM NADH, in 50 mM phosphate buffer pH 7.0; scan rate 15 mV/s; (b): Differantial pulse voltammograms obtained for a cellulose acetate modified with TBO glassy carbon electrode (a) in the absence of NADH and (b) in the presence of 1 mM NADH, in 50 mM phosphate buffer pH 7.0; scan rate 15 mV/s.

The cyclic voltammogram of CA-TBO/GC electrode shows well-defined cathodic and anodic peaks at −283 mV and −240 mV, respectively (Figure 1a) With the addition of 1 mM NADH, the anodic peak current increased and shifted towards a positive direction (−205 mV) whereas the cathodic peak decreased. Following the addition of 1mM NADH, the obtained increase of the anodic peak current can also be seen on the corresponding differential pulse voltammograms (Figure 1b). Finally, NAD^+^ was produced as a result of the reaction between TBO_ox_ and NADH. Then, as a result of the reaction between NAD^+^ and ADH, ethanol was converted to acetaldehyde using the CA-TBO/ADH/GC biosensor. Reactions on the electrode surface occurred according to the following mechanism:

(1) $${\text{TBO}}_{\text{ox}}+\text{NADH}\to {\text{NAD}}^{+}+{\text{TBO}}_{\text{red}}$$

(2) $$\text{Ethanol}+{\text{NAD}}^{+}\overset{\mathrm{ADH}}{\to }\text{Acetaldehyde}+\text{NADH}+H^{+}$$

(3) $${\text{TBO}}_{\text{red}}\overset{\mathrm{electrode}}{\to }{\text{TBO}}_{\text{ox}}+2\bar{e}$$

### 3.3. Effect of enzyme activity on the biosensor response

**Figure 2.**
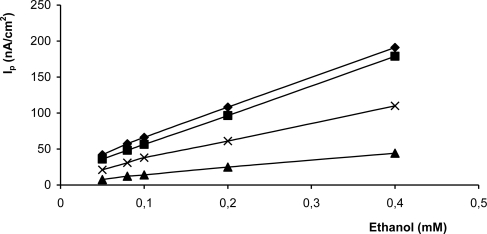
The effect of enzyme amount on the biosensor response (50 mM phosphate buffer; pH 7.0; detection: DPV, scan rate: 15 mV/s, -▴-▴-, 47.1 U cm^−2^, -x-x-, 70,6, -▪-▪-, 117,6 U cm ^−2^, -♦-♦-, 200 U cm^−2^).

To evaluate the effect of the enzyme activity on the biosensor response, different amounts of enzyme were used in the preparation of the biosensor. For this purpose four biosensors containing 200, 117.6, 70.6, 47.1 U cm^−2^ ADH activity were prepared by the immobilization method. While an increase in the ADH activity from 47.1 to 200 U cm^−2^ had a positive effect on the linearity, and also showed higher biosensor responses (Figure 2), an increase in activity from 117.6 to 200 U cm^−2^ didn’t show any clear effect on the biosensor response. Results of 117.6 to 200 U cm^−2^ were very close to each other. The higher amount of ADH in the biomatrix can cause a decrease in peak current sensitivity. The reason why the peak current decreases is the disturbance of the electron relaying capacity at higher enzyme amounts [64]. Because of the high cost of enzymes, 117.6 U/cm^2^ was used in all experiments.

### 3.4. Effect of the temperature on the biosensor response

**Figure 3.**
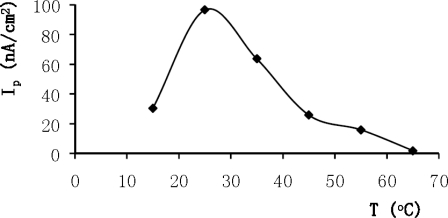
The effect of temperature on the biosensor response (phosphate buffer; pH 7.0, 50 mM, detection: DPV, scan rate: 15 mV/s, 2.0 × 10^−4^ M ethanol).

Since the enzyme activity was dependent upon temperature, the effect of the temperature on the response of the biosensor was investigated for 2.0 × 10^−4^ M ethanol. The range of the temperature was varied from 15 to 65 °C. Experiments were performed in a voltammetric cell filled with 50 mM PB (pH 7.0). The differential pulse voltammograms were recorded in an anodic direction between the ranges of −400 mV and 0 mV with a 15 mV/s scan rate. Results obtained are given in Figure 3. According to the figure, the highest biosensor response was observed at 25 °C. Below and above 25 °C, decreases in the biosensor responses were recorded. There were definite decrease in peak current at higher temperatures due to the deformation of the membrane coated on the electrode surface. Another decrease was also observed below 25 °C because enzyme activity diminished below 25 °C.

### 3.5. Effect of pH on the biosensor response

**Figure 4.**
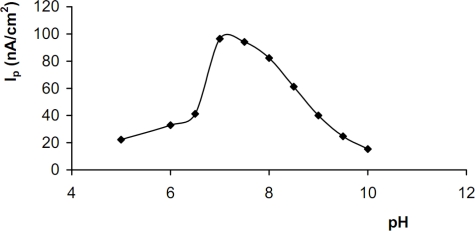
The effect of pH on the biosensor response (2.0 × 10^−4^ M ethanol, T: 25 °C, detection: DPV, scan rate: 15 mV/s).

In order to test the effect of solution pH on the electrochemical behaviour of the biosensor, the voltammetric responses of the biosensor were obtained in 50 mM buffer solutions. The pHs of these buffer solutions varied from 5.0 to 10.0 for 2 × 10^−4^ M ethanol. The experiments were performed with acetate (pH 4.0–5.0), citrate (pH 5.5–6.5), phosphate (pH 7.0–8.5) and glycine (pH 9.0–10.0) buffer solutions. The highest biosensor response was observed at pH 7.0 (Figure 4). In the enzymatic reactions, protons are transferred from one chemical species to another; thus, the solution pH value has a considerable effect on the performance of the prepared ethanol biosensor [65]. Solution pH is a very important parameter for enzyme activity, electron transfer and stability of NAD^+^ and TBO. Therefore, in order to provide optimum stability and activity, pH 7.0 was chosen and used in all the further experiments.

### 3.6. Analytical characterization studies of the biosensor

#### 3.6.1. Operational and long-term stability

**Figure 5.**
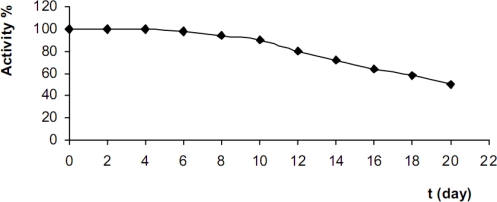
Storage stability of the biosensor (phosphate buffer; pH 7.0, 50 mM, T: 25 °C, detection: DPV, scan rate: 15 mV/s, 2.0 × 10^−4^ M ethanol).

We investigated the operational and long-term stability of the proposed ethanol biosensor. The CA-TBO/ADH/GC was placed in contact with 2.0 × 10^−4^ M ethanol solution in the electrochemical cell in order to study its operational stability under uninterrupted use conditions for 8 h. The current response decreased only about 50% within the first hour, and about 43% within 4 h, which indicated the biosensor has a good operational stability and was stable enough for continuous usage for hours. The long-term stability of the biosensor was investigated by performing triplicate measurements of 2.0 × 10^−4^ M ethanol in phosphate buffer periodically every 2 days for 20 days. When not in use, it was stored at 4 °C in a 50 mM phosphate buffer (pH 7.0). The biosensor lost approximately 50% of its initial sensitivity after two weeks of continual measurements (Figure 5). The sharply response decrease when stored may be attributed to several factors, including the decomposition of membrane, the decomposition of TBO under light, and the inherent instability of ADH [66].

#### 3.6.2. Substrate selectivity

The substrate selectivity of the biosensor was examined for 2 × 10^−4^ M of standards of various substrates such as ethanol, methanol, *n*-butanol and isopropyl alcohol. The results indicated that the biosensor responded to primary aliphatic alcohols, and the maximum responses were obtained for ethanol (Table 2). Compared with the primary aliphatic alcohols, the biosensor response showed significant decrease for branched alcohols. The decrease can be attributed to branched alcohols’ steric effects which make it hard to reach the biosensor surface. Thus, it can be clearly said that the biosensor was very suitable for the ethanol determination.

#### 3.6.3. Linear range of the biosensor

**Figure 6.**
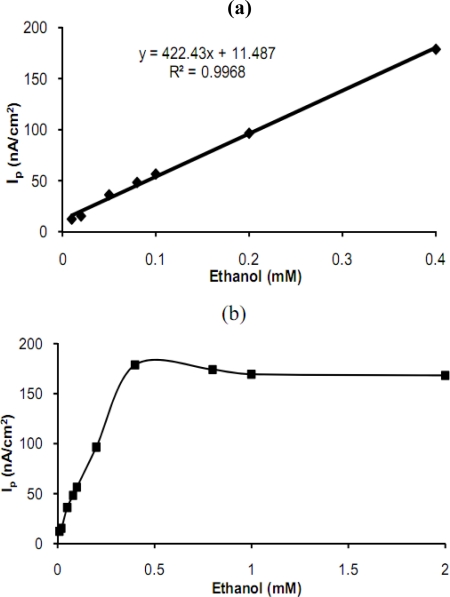
(a) Calibration curve of the biosensor (b) Analytical curve obtained with the biosensor (phosphate buffer; pH 7.0, 50 mM, T: 25 °C, detection: DPV, scan rate: 15 mV/s).

A linear standard curve was obtained in ethanol concentration range between 1 × 10^−5^ M and 4 × 10^−4^ M (*r*^2^ = 0.9968) (Figure 6a). At higher concentrations than 4 × 10^−4^ M, the standard curve showed a deviation from linearity (Figure 6b). The CA-TBO/ADH/GC provides a wider linear range of detection and was more sensitive [7,10] than those of reference systems based on modified biosensors containing catalase and alcohol oxidase enzymes [2,4,5,9]. The detection limit of ethanol, at a signal-to-noise ratio of three, is found to be 5.0 × 10^−6^ M. Comparison of the results with literature data are given in Table 3.

#### 3.6.4. Reproducibility and repeatability

The reproducibility of the experiments was examined for 2.0 × 10^−4^ M ethanol. Eight successive measurements were obtained with a relative standard deviation of 3.2%. The results show that the biosensor can be used to determine ethanol precisely. This electrode-to electrode reproducibility value is better than the modified alcohol dehydrogenase, catalase and alcohol oxidase biosensors [1,3–8]. Also, this modified electrode presented good repeatability, with a relative standard deviation of 4.3% for a series of six measurements of a 2.0 × 10^−4^ M ethanol sample.

### 3.7. Determination of ethanol in sample

To investigate the practical ability, the developed biosensor was employed for the determination of ethanol in commercial beers [light, 3.0% (v/v); dark 6.1% (v/v) and normal 5% (v/v) ethanol] under the optimized conditions. The concentration of ethanol was determined by using the standard addition method due to elimination of matrix effects. The results obtained were 3.0 ± 0.1, 6.1 ± 0.3, 5.0 ± 0.3 vol.% ethanol for light, dark and normal beer, respectively. According to the results, the biosensor could be successfully applied for determination of ethanol in beer samples.

## 4. Conclusions

A novel biosensor based on coimmobilization of TBO, NADH and ADH on a cellulose acetate coated glassy carbon electrode was developed for ethanol determination. There have been no studies in the literature on development of a biosensor based on coimmobilization of TBO, NADH and ADH on cellulose acetate membrane for the determination of ethanol up to now, which make our work original. The covalent linkage of TBO on cellulose acetate membrane leads to a stable electrode material. This material, combined with NADH/ADH system, was very useful as a simple and effective way to develop biosensors for ethanol determination. We characterized the response of the biosensor in terms of the operating pH of the solution, enzyme activity, membrane thickness, substrate specificity, temperature, reproducibility, operational and storage stability. The experiments described above show the ability to use the developed biosensor for the detection of ethanol with good sensitivity, selectivity, and precision desirable for any analytical methodologies. The developed biosensor also exhibits good thermal stability and long-term storage stability as well as showing a low detection limit and rapid response.

## Figures and Tables

**Table 1.** Hydrolysis effect on electrode response.

**Membrane Ratio (% w/v)**	**Peak Current Before Hydrolysis *I_p_* (nA/cm^2^) × 10^3^**	**Peak Current After Hydrolysis *I_p_* (nA/cm^2^) × 10^3^**
1	0.87	4.54
2	0.16	18.2

**Table 2.** Substrate selectivity of the biosensor.

**Substrate**	**Activity (%)**
Ethanol	100
Methanol	40
*n*-Butanol	33
Isopropyl alcohol	3

**Table 3.** Analytical characteristics of ADH based biosensors for ethanol detection.

**Electrode**	**Detection potential (V)**	**Linear range (mM)**	**r**	**Slope (μA/mM)**	**Limit of detection (LOD) (μM)**	**Storage stability**	**References**
ADH-PVA-CNT-GCE	+0.7	up to 1.5	-	0.196	13		64
ADH-MB-CNT-CPE	0.0	0.05–10.0	0.9998	0.597	5		67
ADH-PDDA-CNT-GCE	+0.1	0.5–5.0	0.998	-	90		68
ADH-Au_coll_-MWCNT-Teflon	+ 0.3	0.02–1.0	0.9995	2.27	4.7		69
ADH-MWCNT- Teflon	+0.3	0.10–1.0	0.998	1.8	32		69
ADH-(RuSiNPs)-GCE		1.0 × 10^−4^–10	0.9953	-	0.05	>2 week	70
ADH-MWCNT-CHIT-GCE	+0.7	-	-	0.1646	0.52		65
ADH-AuNPs/PSSG/Ru(bpy)_3_^2+^	-	5.0 × 10^−3^–5.2 × 10^−3^	-	-	12 nM	>one month	71
ADH-PVA-Ru(bpy)_3_^3+^/sol gel	-	0.025–50	-	-	10	2 week	72
BCB/ADH/EG/CCE	0.15	1–13				2 week	57
BCB/ADH/EG/RE	0.15	2–20				2 week	57
NB/ADH/EG/CCE	0.15	1–22				2 week	57
NiHCF/ADH/Au	0.55	Up to 5			0.5	1 week	3
SIRE/ADH	0.95	0–12.5	0.9874		<0.1	48 hour	6
SNMB/ADH/GP	0.0	0.1–10	0.9996		8	3 month	7
DA/ADH/EG/CCE	0.15	1–4				4 month	29
PVA/MWCNT/ADH/GCE	0.6	Up to 1.5				7 days	64
Ru-AuNPs/ADH/ITO		0.01–10	0.993		3.33	2 week	73
MB/MWCNT/ADH/GP	0.0	0.05–10	0.9998		5		67
CA-TBO /ADH/ /GCE	−0.4–0	0.01–0.4	0.9968	0.41	5	2 week	This work

ADH;Alcohol dehydrogenase, PVA; Poly (vinyl alcohol), CNT; Carbon nanotube, GCE;Glassy carbon electrode, MB; Meldola’s Blue;

PDDA; Poly (dimethyl diallyl ammonium chloride), MWCNT; Multiwall carbon nanotube, CHIT;Chitosan, PSSG; The partial sulfonated (3-mercaptopropyl)-trimethoxysilane-sol-gel, RuSiNPs; Ru(bpy)_3_–doped silica nanoparticles, NB;dye, BCB;dye, EG;Exfoliated graphite, RE; Recompresed electrodes, CCE; Ceramic carbon electrode, NiHCF;Nickel hexacyanoferrate, SIRE; Sensors based on injection of the recognition element, DA; Dopamine, ITO;Indium tin oxide, Ru-AuNPs-Ru(bby)_3_–AuNPs aggregates; ECL;Electrogenarated chemiluminescence, GP;Graphite powder.
